# Increased pore size of scaffolds improves coating efficiency with sulfated hyaluronan and mineralization capacity of osteoblasts

**DOI:** 10.1186/s40824-019-0172-z

**Published:** 2019-12-18

**Authors:** Jan Krieghoff, Ann-Kristin Picke, Juliane Salbach-Hirsch, Sandra Rother, Christiane Heinemann, Ricardo Bernhardt, Christian Kascholke, Stephanie Möller, Martina Rauner, Matthias Schnabelrauch, Vera Hintze, Dieter Scharnweber, Michaela Schulz-Siegmund, Michael C. Hacker, Lorenz C. Hofbauer, Christine Hofbauer

**Affiliations:** 1 Institute for Pharmacy, Pharmaceutical Technology, University Leipzig, Leipzig, Germany; 2 Division of Endocrinology, Diabetes, and Metabolic Bone Diseases, Department of Medicine III, Technische Universität Dresden, Fetscherstraße 74, 01307 Dresden, Germany; 3 Center for Healthy Aging, TU Dresden Medical Center, Dresden, Germany; 4 Institute of Materials Science, Max Bergmann Center of Biomaterials, Technische Universität Dresden, Dresden, Germany; 5 Present address: Department of Cellular and Molecular Medicine, Glycobiology Research and Training Center, University of California, San Diego, La Jolla, CA USA; 6 Leibniz Institute of Polymer Research Dresden, Dresden, Germany; 7 Biomaterials Department, INNOVENT e.V, Jena, Germany; 8 Center for Regenerative Therapies, Dresden, Germany; 9 Orthopedics and Trauma Surgery Center, Technische Universität Dresden, Dresden, Germany

**Keywords:** Biomaterial, Macroporous scaffold, Mineralization, Osteoblast differentiation, Poly(lactic acid), Sulfated glycosaminoglycan

## Abstract

**Background:**

Delayed bone regeneration of fractures in osteoporosis patients or of critical-size bone defects after tumor resection are a major medical and socio-economic challenge. Therefore, the development of more effective and osteoinductive biomaterials is crucial.

**Methods:**

We examined the osteogenic potential of macroporous scaffolds with varying pore sizes after biofunctionalization with a collagen/high-sulfated hyaluronan (sHA3) coating in vitro*.* The three-dimensional scaffolds were made up from a biodegradable three-armed lactic acid-based macromer (TriLA) by cross-polymerization. Templating with solid lipid particles that melt during fabrication generates a continuous pore network. Human mesenchymal stem cells (hMSC) cultivated on the functionalized scaffolds in vitro were investigated for cell viability, production of alkaline phosphatase (ALP) and bone matrix formation. Statistical analysis was performed using student’s t-test or two-way ANOVA.

**Results:**

We succeeded in generating scaffolds that feature a significantly higher average pore size and a broader distribution of individual pore sizes (HiPo) by modifying composition and relative amount of lipid particles, macromer concentration and temperature for cross-polymerization during scaffold fabrication. Overall porosity was retained, while the scaffolds showed a 25% decrease in compressive modulus compared to the initial TriLA scaffolds with a lower pore size (LoPo). These HiPo scaffolds were more readily coated as shown by higher amounts of immobilized collagen (+ 44%) and sHA3 (+ 25%) compared to LoPo scaffolds. In vitro, culture of hMSCs on collagen and/or sHA3-coated HiPo scaffolds demonstrated unaltered cell viability. Furthermore, the production of ALP, an early marker of osteogenesis (+ 3-fold), and formation of new bone matrix (+ 2.5-fold) was enhanced by the functionalization with sHA3 of both scaffold types. Nevertheless, effects were more pronounced on HiPo scaffolds about 112%.

**Conclusion:**

In summary, we showed that the improvement of scaffold pore sizes enhanced the coating efficiency with collagen and sHA3, which had a significant positive effect on bone formation markers, underlining the promise of using this material approach for in vivo studies.

## Introduction

Primary and secondary osteoporosis caused by e.g. long term glucocorticoid application or diabetes mellitus are highly prevalent diseases in the ageing society. Worldwide almost 390 million people aged over 50 suffer from low bone mass and strength, resulting in increased fracture risk [1, 2]. In addition, these patients often display delayed fracture healing which leads to a persistent immobility and the need for special care [1].

Osteoporosis is the result of an imbalance between bone formation and bone resorption. Osteoporotic patients commonly show a reduced activity of bone forming osteoblasts with a decreased differentiation capacity and reduced bone matrix synthesis that does not match bone resorption by osteoclasts. The understanding of the pathogenesis of osteoporosis led to remarkable advances in the development of treatment strategies and disease prevention [3, 4]. However, as osteoporosis does not present with overt symptoms in its early stages, the disease remains highly underdiagnosed. Often patients are only diagnosed after a fragility fracture. At this stage detrimental bone changes are far more advanced and pose a challenge. Whereas the underlying disease can be managed long term with e.g. anti-resorptive or osteo-anabolic drugs, bone healing and/or osseo-integration at a fracture site have different needs. Here, local stimuli need to act in a more potent manner to ensure enhanced bone formation and proper bone healing. Therefore, it is of utmost importance to discover new and potent local therapy options [5–8].

For bone replacement, autografts remain the gold standard. This strategy is, however largely limited by graft availability and donor site morbidity [9, 10]. In order to fulfill the needs of patients with compromised bone healing capacity biomaterial design has progressed from the first^,^ mainly inert biomaterial generation, to bioactive and biodegradable second-generation materials and now finally to cell instructive third generation materials [11]. Besides combining the characteristics of the first two generations such as providing mechanical support and stimulating osteo-conductivity these materials provide a microenvironment stimulating osteogenesis and bone healing. This can be achieved by loading the biomaterials with special molecules such as growth factors, hormones, or chemicals [9]. Materials were designed to directly modulate the activity of osteoblasts by loading bone morphogen proteins (BMPs) on different scaffold types or by creating an anti-inflammatory environment via an injectable heparin-based and cytokine loaded microsphere under diabetic conditions. The basic material itself is often an inorganic bone substitute, such as calcium phosphate (CaP) ceramics, or an organic material, such as one of the manifold variations of polymers like polyethylene glycol (PEG) or poly (lactic acid) (PLA) [5]. These materials can be adjusted in composition, porosity, and stiffness to meet the requirements regarding biodegradability and material strength.

In recent years, glycosaminoglycans (GAGs), which are a major component of the organic compartment of the extracellular matrix (ECM) in bone, have been shown to exert positive effects on the regenerative potential of bone cells. Hyaluronan (HA) and chondroitin sulfate (CS) support the osteogenic action of osteoblasts [12, 13] while concurrently suppressing differentiation and resorption activity of osteoclasts [14, 15] in a sulfation degree-dependent manner. These effects are mediated directly or indirectly by altering the gene expression of osteocytes, the local orchestrators of bone remodeling, towards an osteo-anabolic direction [16]. Additionally, it was shown that sulfated GAG (sGAGs) have the ability to bind key players of bone remodeling such as osteoprotegerin (OPG), BMP-2 and the potent Wnt inhibitor sclerostin and change their bioactivity [17]. This effect is mediated by a heparin-binding domain that also conveys the effects of other GAGs on these molecules with the synthetically derived high-sulfated hyaluronan (sHA3) as the most potent binder [18, 19].

As sGAGs showed potent osteo-inductive potential in vitro, they were also tested in vivo to analyze their effect on bone healing. In healthy rats, a non-osteoinductive material coated by an artificial ECM (aECM) consisting of collagen and sHA3 displayed enhanced bone healing [20]. In addition, also in type 2 diabetic rats the healing of bone defects could be improved by using biodegradable lactic acid-based (TriLA) scaffolds coated with collagen and sHA3 caused by an increased osteoblast activity and sequestration of sclerostin in the defect area [19]. The TriLA scaffolds were composed of the biomaterial Tri134LA6, part of a recently developed platform of biodegradable macromers [21]. The TriLA macromer platform consists of a trivalent alcohol core which is modified by biodegradable poly (lactic acid, LA) oligoesters (6 LA per arm for Tri134LA6). The three arms are terminated by methacrylates for cross-(co-)polymerization. Thermally induced polymerization in the presence of partially molten lipid particles enables the generation of porous scaffolds from the material [21]. Cross-co-polymerization of PEG monomethacrylate is used to incorporate free functionalities for additional covalent modification, [22] and PEG addition was recently found to also positively affect bone mineralisation behaviour in LA-based porous scaffolds [23]. For porous scaffolds, general porosity, pore size and pore interconnectivity are three parameters that contribute to the cellular responses to the material [24, 25]. Suitable pore sizes for bone formation purposes were found to be in excess of 300 μm, to enable sufficient vascularisation of the material and prevent hypoxic conditions in the inner regions [26, 27]. This is consistent with our observation using non-cross-polymerized PLGA (poly (lactic acid-*co*-glycolic acid) scaffolds [27]. Here, pore sizes ranging from 300 to 500 μm yielded the best results with respect to collagen production, hydroxyapatite deposition and bone mineral maturation. Newly synthesized matrix on scaffolds with a lower or higher mean pore size contained less collagen and mineral and was less mature. Because the healing of the bone gap defects in our diabetic rat study was improved, but not completed by the use of coated TriLA scaffolds, the material properties were a factor thought to be an avenue for optimization.

In this study, we adjusted the pore size of TriLA scaffolds to a one hypothetically more suitable for bone formation purposes by varying the scaffold fabrication parameters. Subsequently, the scaffolds were coated with aECM containing sHA3 to investigate the osteoanabolic effect on osteoblasts in vitro and elucidate the osteogenic potential of these high pore size scaffolds as a balance of better nutrient supply but decreased surface area resulting from the increased pore size and reduced mechanical properties. We determined that elevation of pore size increased osteogenic potential of osteoblasts.

## Materials and methods

### Solid lipid microsphere preparation

The solid lipid microspheres were prepared as described previously [28]. In brief, 10 g of the lipids Softisan 154 (melting range: 53–58 °C, Sasol, Germany) and Witepsol H37 (melting range: 36–38 °C, Sasol, Germany) were mixed with 7.5 g water in the desired ratio (shown as Softisan:Witepsol) and melted at 65 °C in a polypropylene tube. Following emulsification by 20-fold inversion of the tube, the dispersion was cast into 600 mL water and stirred for 5 min at 900 rpm and 15 °C. The resulting particles were collected by filtration, rinsed with cold water (15 °C) and spread on filter paper to dry for 2 days. Particles with a size between 300 and 500 μm were separated by sifting.

### TriLA-scaffold generation

As described previously, biodegradable three-armed methacrylate-terminated macromers (Tri134LA6) were synthesized from trimethylolpropane (MW 134 Da, 1 eq, Sigma-Aldrich), D,L-lactide (9 eq, for a theoretical incorporation of 6 lactic acid units (LA) per arm, Sigma-Aldrich) and methacryloyl chloride (3.75 eq, Sigma-Aldrich) [21]. For generation of TriLA/polyethylene glycol scaffolds, the oligomer and PEG-monomethacrylate (PEG-MA, MW 1000 Da) (ratio 5:1 by weight) were dissolved in acetone/dichloromethane (5:3, V/V) and cross-polymerized thermally at a constant temperature set to value between 50 and 58 °C depending on the formulation, using benzoyl peroxide (Sigma-Aldrich) and 4-(*N,N*-dimethylamino) phenethyl alcohol (Sigma-Aldrich) as the initiator system.

For the generation of macroporosity, cross-polymerization was carried out in the presence of the solid lipid microspheres (size fraction: 300 to 500 μm). The amount of microspheres ranged from 1.5 to 2.75 times the combined mass of oligomer and PEG-MA. Following polymerization, the lipid was removed from the scaffolds using n-hexane and isopropanol, and the macroporous cylinders were trimmed to the desired size using biopsy punches and razor blades. The dried scaffolds were then stored under vacuum.

For cell culture experiments, scaffolds were sterilized using gamma radiation (15 kGy, at Synergy Health, Radeberg, Germany).

### Mechanical characterization

In order to determine biomechanical properties we analyzed the compressive modulus and compressive strength of scaffolds employing a Shimadzu EZ Test universal testing apparatus equipped with a 100 N load cell (Hegewald & Peschke, Nossen, Germany). Therefore, samples of cylindrical shape with a height of 3 mm and a diameter of 5 mm were subjected to mechanical stress at a crosshead speed of 1 mm/min. The resulting force-strain graphs were used to calculate compressive strength and compressive modulus, employing the WinAGS Lite Software provided with the testing apparatus.

### Visualization by stereo microscopy and scanning electron microscopy (SEM)

Cylindrical scaffold samples with a height of 3 mm and a diameter of 5 mm were examined under a stereo microscope (SM33, Hund Wetzlar, Wetzlar, Germany) and documented with a Nikon camera (DS-2Mv) using the NIS-Elements software (Nikon, Duesseldorf, Germany).

High-resolution pictures were taken via SEM. To this end scaffold disks with a thickness of 0.5 to 1 mm were applied to a sample holder using Conductive Carbon Cement (Leit-C, Plano, Wetzlar, Germany) and coated with gold (Sputter coater MED 020, Bal-Tec, Leica Microsystems, Wetzlar, Germany). Images were recorded on a CS 44 scanning electron microscope (Cam Scan, Cambridgeshire, United Kingdom) with the Noran System Six software (version 1.8).

Scanning electron microscopy of hMSC on the scaffolds was carried out using a Zeiss DSM 982 Gemini FESEM (Oberkochen, Germany) to assess cell morphology. For sample preparation, hMSC were seeded on coated scaffolds and cultivated for 3 days. Subsequently, cells were fixed with 4% paraformaldehyde and dehydrated using a graded ethanol series followed by infiltration with hexamethyldisilazane (Fluka, Germany). Samples were then mounted on stubs and coated with carbon in a Bal-Tec SCD 050 coater (Bal-Tec AG, Liechtenstein). Microscopy was performed in HiVac mode at an acceleration voltage of 3 kV using the SE-Inlens detector.

### Preparation of modified HA derivatives

Low molecular weight HA (LMW-HA) was prepared by ozonolysis of high molecular weight native HA as previously described [29]. The high-sulfated hyaluronan derivative sHA3 was synthesized by sulfation of LMW-HA with SO_3_-DMF and characterized as previously reported [29, 30]. Analytical data of the prepared HA derivatives (LMW-HA, sHA3) are summarized in Table 1.

**Table 1:** Analytical data of synthesized GAG derivatives

Sample	LMW-HA	sHA3
DS	–	3.0
Mn [g mol^−1^]	28,130 (84000)	29,900 (52800)
Mw [g mol^− 1^]	57,270 (91200)	62,000 (93700)
PD	2.4	1.8

*LMW-HA* low molecular HA, *sHA3* high-sulfated HA, *DS* average number of sulfate groups per repeating disaccharide unit, *Mn* number-average molecular weight, *Mw* weight-average molecular weight, analyzed by gel permeation chromatography (GPC); values as determined with Laser Light Scattering detection and Refraction index detection (in parentheses), *PD* polydispersity index detected by GPC; values calculated from RI detection

### aECM coating of scaffolds

TriLA scaffolds with different pore sizes (fabricated with reference parameters LoPo and optimized parameters HiPo) were coated with collagen-based aECMs with or without sHA3 under sterile conditions as described previously [19]. In brief, TriLA scaffolds were wetted in Dulbecco phosphate-buffered saline (PBS, pH 7.4) applying partial vacuum to force the infiltration of the solution into the porous scaffold architecture. Afterwards, the scaffolds were incubated under partial vacuum overnight at 37 °C in a 1:1 volume mixture of 2 mg/ml acid-solubilized rat tail collagen type I (Corning, Kaiserslautern, Germany) and fibrillogenesis buffer (60 mM phosphate buffer, pH 7.4), containing 3.918 mg/ml sHA3 in case of the collagen/sHA3 coatings. Then, the scaffolds coated with collagen or collagen/sHA3 were freeze-dried prior to washing for two times with double distilled water (ddH_2_O) under partial vacuum conditions. Non-coated scaffolds, which were wetted but not incubated with an aECM coating dispersion, served as control. Prior to any in vitro cell culture experiments, all scaffolds were incubated at 37 °C for 60 min in PBS to rehydrate the aECM coatings.

### Characterization of scaffold pore size and pore size distribution

The mean pore size and the pore size distribution of LoPo and HiPo scaffolds (*n* = 3) were investigated with micro-computed tomography (scanner: vivaCT 75, Scanco Medical, Brüttisellen, Switzerland). For all samples 1000 radiographic images (image resolution: 20.5 μm, X-ray energy: 45 keV) were obtained. Pore size and pore size distribution of the reconstructed μCT-data were analyzed with Scanco evaluation software. The 3D visualization of scaffold data was performed with VG Studio Max 2.2 (Volume Graphics, Heidelberg, Germany).

### Characterization of collagen and sHA3 coating stability on TriLA scaffolds

For evaluation of the stability and distribution of collagen-based aECM coatings on the TriLA scaffolds, the remaining collagen and sHA3 contents were analyzed after incubation of the scaffolds in PBS at 37 °C for 60 min as well as after an additional incubation at 4 °C for 7 days or at 37 °C for up to 14 days. The collagen content of the coatings was visualized by staining with Sirius red dye (0.1% solution in picric acid). Excessive dye was removed by washing with 0.01 M hydrochloric acid. Collagen was quantified by measuring the fluorescence intensity (λ_ex_ = 340 nm, λ_em_ = 440 nm) relative to a collagen calibration after digestion of the coatings with collagenase (Sigma-Aldrich, Schnelldorf, Germany, 0.0125 mg/ml in TES buffer, pH 7.4) at 37 °C for 16–18 h and reaction with Fluoraldehyde *o*-phthaldialdehyde solution (Thermo Fisher Scientific, Schwerte, Germany) [19]. Toluidine blue staining was used to visualize the presence of sHA3 within the coating. Therefore, the scaffolds were incubated in 0.4 mg/ml Toluidine blue (Sigma-Aldrich, Schnelldorf, Germany) dissolved in 0.1 M hydrochloric acid with 2 mg/ml sodium chloride for 240 min. A prior washing step with ddH_2_O removed non-bound Toluidine blue. In order to assess potential differences in the sHA3 contents between the studied TriLA porosities, the amount of sHA3-bound dye was re-dissolved from the coatings in 0.02 M sodium hydroxide in 80% ethanol solution for 90 min under constant shaking and the absorbance was measured at 530 nm.

### Cell culture on TriLA scaffolds

Over a time period of 18 days, pre-osteoblastic, murine MC3T3-E1 cells and hMSC were cultured in osteogenic medium (α-MEM, Biochrom, Germany) containing 10% fetal calf serum (Biochrom AG), 1% penicillin/streptomycin (PAA, Germany), 2 mM L-glutamine (PAA), 100 μM dexamethasone (Sigma-Aldrich, Germany), 10 mM β-glycerol phosphate (Sigma-Aldrich), 100 μM ascorbate phosphate (Sigma-Aldrich), and 5 ng/ml BMP-2 (PeproTech, Germany) at 37 °C and 5% carbon dioxide [31]. Therefore, 8 μl of cell suspension, consisting of the cell culture medium and 100.000 cells, was applied on each scaffold and incubated for 20 min to allow cell attachment to scaffold surface. Afterwards, wells were filled up with cell culture medium until the scaffolds were completely covered. Every other day, the scaffold was transferred into a new well and the cell culture medium was changed. Culturing hMSC was approved by the ethics committee of the Faculty of Medicine of the Technische Universität Dresden (EK 245082010).

### Cell viability test

For determination of the cell viability 100.000 cells per scaffold were seeded and cultivated up to 18 days. Then 10 μl CellTiterBlue® Reagent (Promega, USA) per 100 μl cell culture medium was added. After an average time of 2 h, cell culture supernatant was transferred into a black 96-well plate to measure fluorescence intensity (560–590 nm, Fluostar Omega, BMG Labtech, Germany), which increases with enhanced metabolic activity of mitochondria.

### Histology analysis of scaffold mineralization and cell number

After 18 days in culture, scaffolds were fixed for 1 h in 4% PFA and dehydrated using rising ethanol series from 50 to 100% ethanol concentration. Scaffolds were embedded in paraffin (Leica Biosystems, USA) and slices of 4 μm were prepared with a Microtome 2265 (Leica). These slices were then stained with *von Kossa* that visualizes mineralized matrix. The mineralization capacity was quantified with standard bone histomorphometry using OsteoMetrics OsteoMeasure™ software (OsteoMetrics, USA) [32]. In brief, 15 fields-of-view were defined throughout the scaffold as crossing lines to ensure uniform analysis of the whole scaffold and in between samples. In each field-of-view, the mineralized area was then manually outlined and related to the total area of the field-of-view.

### ALP activity

After 7 days in culture, the scaffolds were washed with PBS and subsequently lysed for 15 min with 50 μl ALP lysis buffer (Triton X-100, protease inhibitor) at RT on a shaker. The supernatant was centrifuged at 25.000 g at 4 °C for 30 min. For ALP determination, 10 μl of the supernatant were diluted in 90 μl ALP sample buffer containing *p*-nitrophenol for 30 min at 37 °C. ALP enzyme activity can then be quantified by resulting yellow color change measured at 405 nm. Enzyme activity was then normalized to the total protein content. Therefore, sample protein concentration was measured using BCA Kit (Peirce, Thermo Scientific) at 37 °C for 30 min.

### ALP staining

Scaffolds were washed with PBS, fixed for 30 s with Aceton-Citrate Solution (60% acetone, 40% 1:50 diluted Citrate Solution (Sigma)), and washed with ddH_2_O. Staining solution (Fast Violet B salt Grade B + Naphtol AS mix (Sigma)) was added to the scaffolds for 30 min at RT in the dark.

### Cell proliferation

To assess the effect of the different pore sizes on cell proliferation LoPo and HiPo scaffolds with a collagen/sHA3 coating were seeded with 100.000 cells/scaffold. After 24 h and 72 h cell quantities were measured using the Quant-iT™ PicoGreen™ dsDNA Assay Kit (Invitrogen) according to the manufactures protocol. Sample cell numbers were calculated from a standard curve of cell lysates with defined hMSC numbers.

### Statistics

The differences in porosity and pore size were analyzed by a student’s t-test and the coating efficiency by two-way ANOVA. For analysis of the effect of HiPo scaffolds and coating (coll and coll/sHA3) on osteoblast activity and differentiation, a two-way ANOVA with Tukey’s post-hoc test was performed using GraphPad Prism 6.0 software. The results are given as mean ± standard deviation (SD). *P* values of < 0.05 were considered statistically significant.

## Results

### Porosity adjustment

**Fig. 1:**
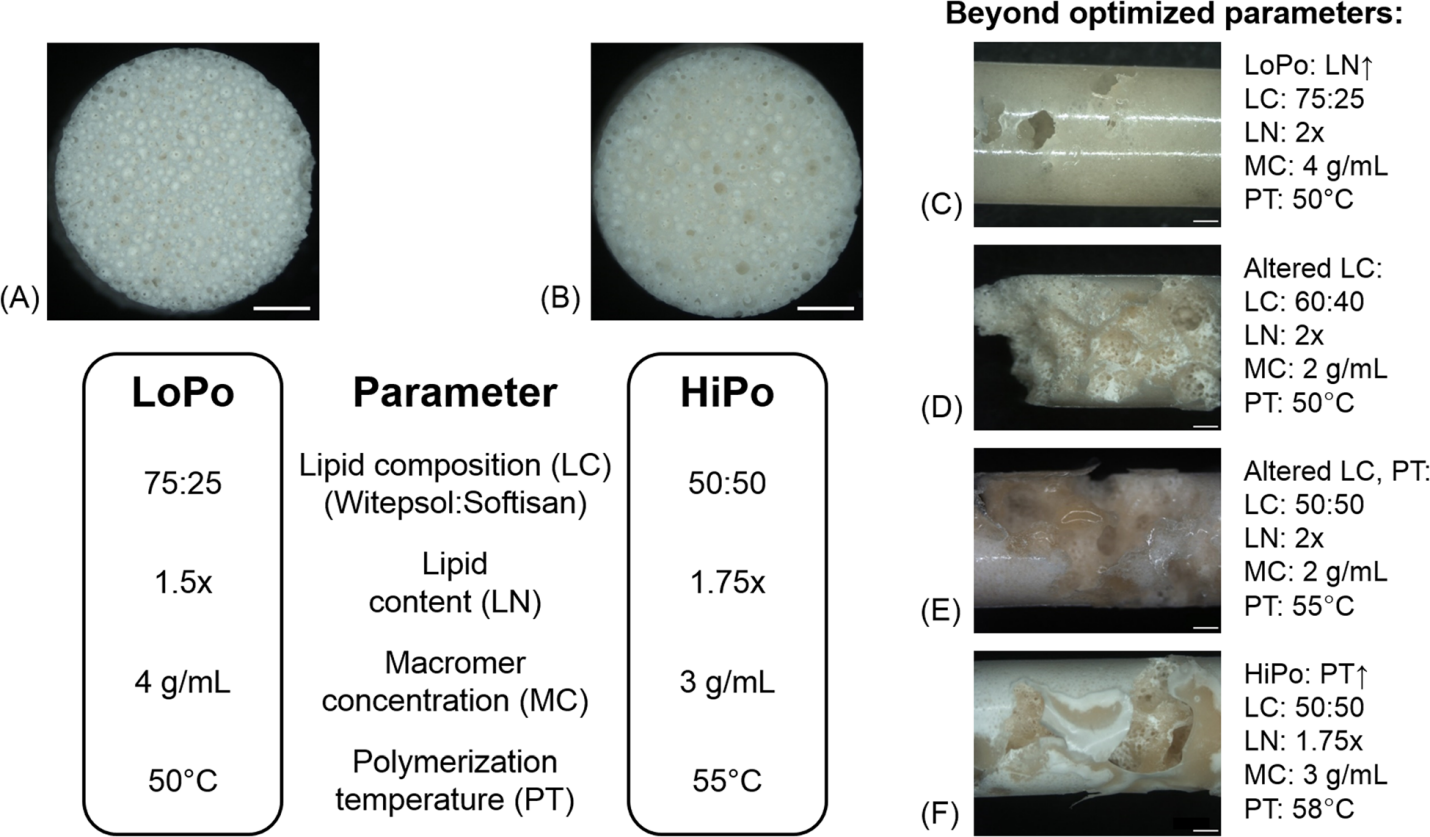
Summary of key process parameters before and after process optimization to enhance scaffold pore sizes. Cross-sections of the (**a**) initial LoPo (low porosity) and ( **b**) optimized HiPo (high porosity) formulation are shown. Lipid content given as mass relative to combined mass of macromer and PEG-MA. (**c**-**f**) Lateral view of representative scaffold produced beyond the optimized values. (**c**) 2x Lipid (75:25 – high:low melting range), shows effect of solely increased lipid amount; 4 g/mL, 50 °C; (**d**) 2x Lipid (60:40), 2 g/mL, 50 °C, shows effect of the improved lipid/macromer amounts with lower melting range particles; (**e**) 2x Lipid (50:50), 2 g/mL, 55 °C, shows effect of improved lipid/macromer amounts with increased temperature; (**f**) 1.75x Lipid (50:50), 3 g/mL, 58 °C, shows effect of optimized formulation with too high temperature. Further details regarding the tuning were compiled in the supporting information. Scale bar = 1.000 μm

As reference material, porous TriLA scaffolds made from the Tri134LA6 macromer were fabricated employing the parameter set and porogen fraction (300–500 μm) established during the development of the TriLA material platform (Fig. 1a, LoPo). In order to potentially improve scaffold pore characteristics like pore interconnect size, the aim was to investigate the effect of adjusting porogen composition, porogen content, macromer concentration and polymerization temperature regimen. Following polymerization, the success or failure of the tested combinations was assessed visually by the generation of an intact, non-perforated scaffold cylinder. Ultimately, a set of optimized parameters was identified (Fig. 1b, HiPo).

During scaffold fabrication macromer cross-co-polymerization as well as lipid particle melting and extraction had to be carefully orchestrated in order to achieve a homogeneous network of interconnected pores [21]. This interplay is significantly affected by macromer concentration of the macromer/PEG-MA solution and the lipid content. Consequently, variations of these parameters had to be evaluated to modify pore network structure. At the reference macromer concentration of 4 g/mL, an increase in lipid content resulted in inhomogeneous pore structure (Fig. 1c). Lowering the macromer concentration to 2 g/mL while increasing the lipid content to two times macromer/PEG-MA mass resulted in intact scaffold cylinders. Further reduction of the macromer concentration below 2 g/mL or increasing the lipid content above two times the combined mass of macromer and PEG-monomethacrylate (PEG-MA) did generate large defects in the cross-polymerized cylinders (Additional file 1: Figure S1).

The melting range of the lipid porogen is a function of their composition and their respective melting ranges of their constituent lipids. The lipid particles employed in scaffold fabrication were composed of two commercially available solid lipids, one with a higher melting range of 53–58 °C (Softisan 154) and one with a lower melting range of 36–38 °C (Witepsol H37). Increasing the content of the lipid component with the lower melting range would result in a faster melting of the microspheres at polymerization temperature and at a higher fraction of melted lipid phase at any time point of the copolymerization process. A faster melting of the lipid particles was suspected to result in an improved pore network. A macromer concentration of 2 g/mL and lipid content of two times macromer/PEG-MA mass resulted in intact scaffold cylinders with the reference particles (75% Softisan:25% Witepsol) (Additional file 1: Figure S1). The same formulation with lipid particles with a higher content of low melting range lipid only produced perforated cylinders. An increase of the lower melting lipid from 25 to 40% did also not yield an intact pore network (Fig. 1d). This was also observed for higher concentrations of the lower melting lipid (Additional file 1: Figure S2). These results indicate that with this macromer concentration and lipid content, network cross-polymerization was not fast enough to yield intact networks.

Temperature-dependent radical-based polymerization like the one used for cross-polymerization of the TriLA-macromer can be accelerated by higher reaction temperatures, which, however, would also result in higher lipid melting rates. With the macromer concentration of 2 g/mL and lipid content of two times macromer/PEG-MA mass, increasing the polymerization temperature from 50 °C to 55 °C still failed to produce undamaged scaffold cylinders (Fig. 1e). Increasing macromer concentration to 3 g/mL without changing lipid content likewise resulted in failure, while increasing macromer concentration in concert with lowering lipid content gave undamaged scaffold cylinders (Additional file 1: Figure S3). Further increase of the polymerization temperature did not result in a successful generation of scaffold cylinders (Fig. 1f).

Following these pilot study, an optimized formulation was established (Fig. 1b, HiPo). Compared to the reference formulation, the optimized scaffolds are produced at a higher processing temperature of 55 °C, with a lower macromer concentration of 3 g/mL, a lipid microsphere content of 1.75 times the mass of macromer/PEG-MA and the microsphere featuring a composition of equal parts high melting range and low melting range lipid. Attempts to combine the optimized formulation with bigger lipid microspheres (500–710 μm) to further increase pore size were not successful (Additional file 1: Figure S3).

### Elevated mean pore size in HiPo scaffolds

**Fig. 2:**
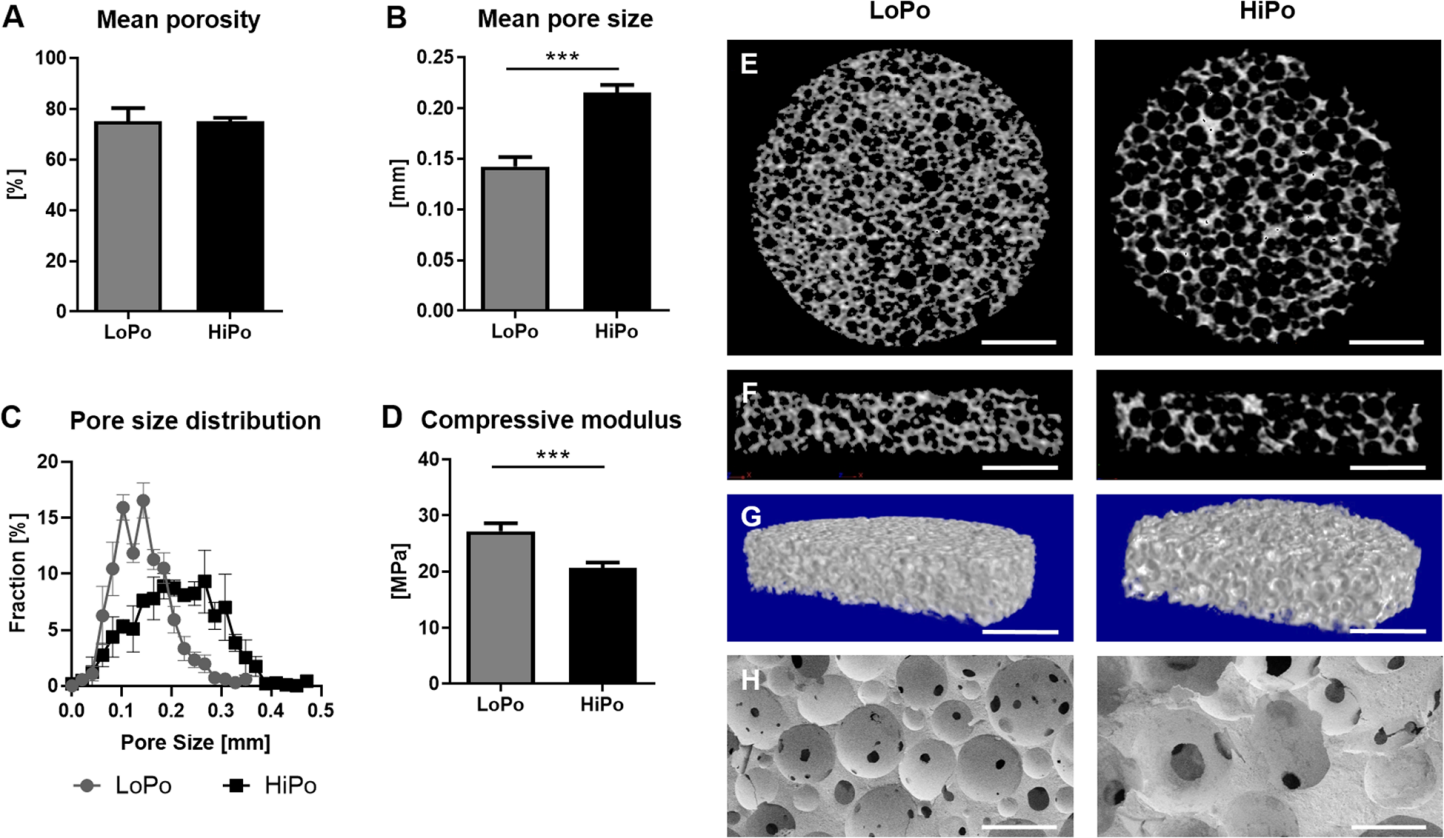
Characterization of LoPo and HiPo scaffold formulations. Using micro-computed tomography, **a** the mean porosity, **b** mean pore size, and **c** amount of scaffold pores per pore size of LoPo and HiPo (low and high porosity, respectively) scaffolds was analyzed. **d** The stability of scaffolds was evaluated by compression testing. **e** Cross and **f** longitudinal section are presented as well as (**g**) 3D visualization and (**h**) REM images of the scaffolds (scale bars: E: 1100 μm, F: 1050 μm, G: 1.500 μm, H: 250 μm). Data represent the mean ± SD. Statistical analysis was performed by student’s t-test. ***: *p* < 0.001

Using micro-computed tomography, the porosity and pore size distribution of the scaffolds were analyzed. While the mean porosity did not differ between the scaffold types, the mean pore size was significantly increased (+ 34%) in the optimized scaffolds (Fig. 2. A, B, E-H). Scaffolds fabricated with the reference parameters had a higher number of smaller pores ranging from 100 to 200 μm and will thus be referred to as LoPo (low pore size), whereas scaffolds with the optimized fabrication parameters show a broader distribution with respect to their pore size (Fig. 2c) and will thus be referred to as HiPo (high pore size). In addition, it was also demonstrated that the mechanical stability, detected as compressive modulus, of HiPo scaffolds was decreased compared to the LoPo scaffolds (− 25%, Fig. 2d).

### Increased immobilization efficiency on HiPo scaffolds

**Fig. 3:**
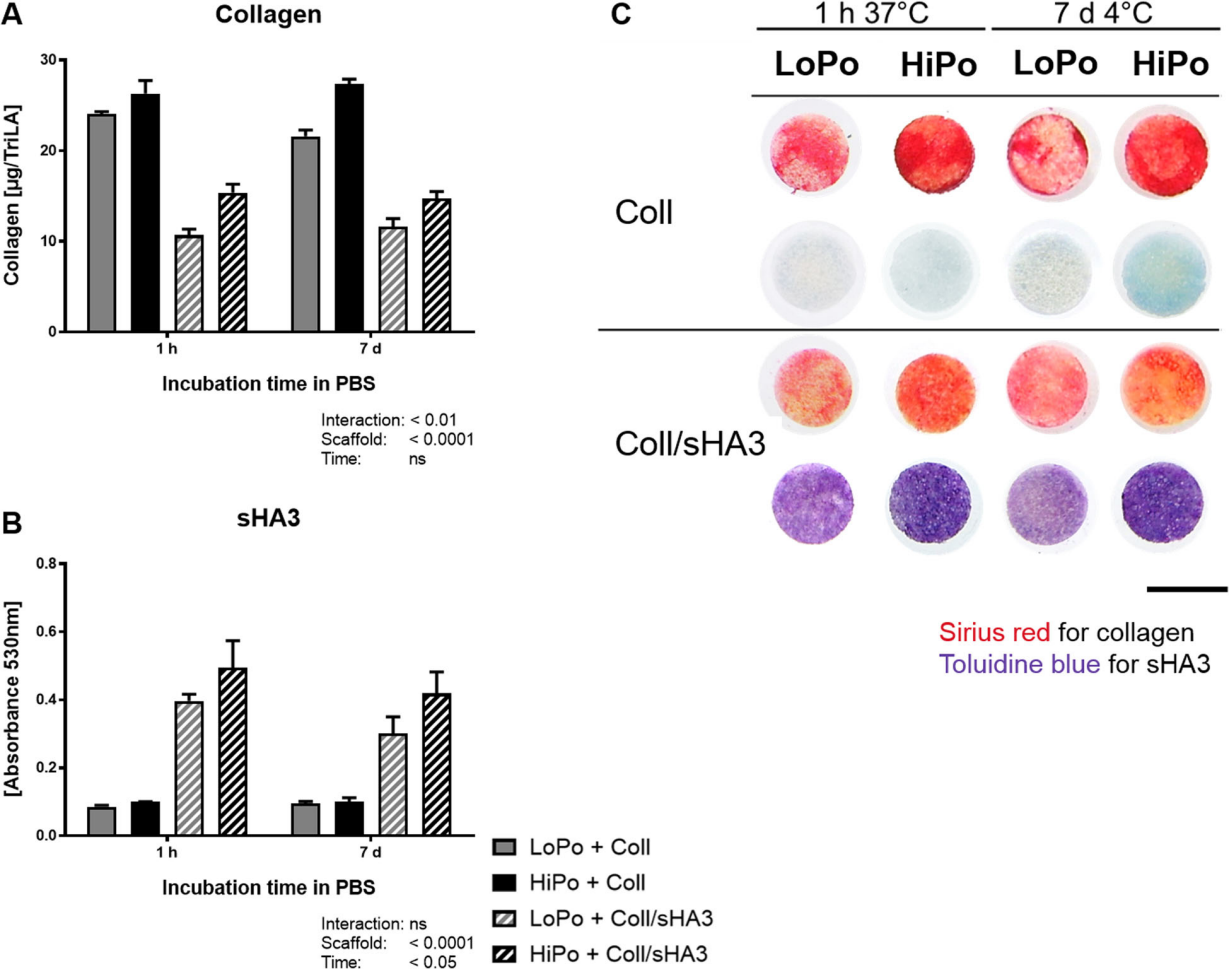
Characterization of aECM modification on scaffolds. The amount of collagen (coll) and high-sulfated hyaluronan (sHA3) immobilized on low (LoPo) and high porosity (HiPo) scaffolds was detected after one hour at 37 °C and after 7 days at 4 °C. **a** Collagen content was analyzed by *o-*phthaldialdehyde (OPA) assay and **b** amount of bound sHA3 by Toluidine blue assay. **c** Qualitative analysis of collagen and sHA3 was performed using Sirius red and Toluidine blue, respectively. Data represent the mean ± SD. Statistical analysis was performed by two-way ANOVA for the effect of scaffold and time and the interaction (scaffold*time). Scale bar: 5 mm

In order to test the efficiency of scaffold coating, the amounts of coll and sHA3 were assessed after one hour at 37 °C mimicking physiological conditions. The HiPo material characteristics increased the amount of collagen on the surface when applied alone (+ 9%), or in combination with sHA3 (+ 44%, Fig. 3 A, C). Also, a higher absorbance for Toluidine blue, which indicates increased amounts of dye-binding sHA3 within the aECM was detected on HiPo scaffolds (+ 25%) compared to LoPo (Fig. 3 b, c). As previously shown, combined coating of coll and sHA3 resulted in a reduced coating efficiency for collagen compared to single collagen coating [19]. Medical devices should be storable for at least one week. We therefore also assessed the coating stability after a simulated 7 day storage period in PBS at 4 °C. The coatings with coll and sHA3 were stable on both scaffold types and the highest fraction of retained coating was detected on HiPo compared to LoPo scaffolds (coll: + 27%, sHA3: + 39%) (Fig. 3 a-c). After 14 days at 37 °C 38–59% of coll and 47–57% of sHA3 content were still present compared to the amount after 1 h of incubation in PBS for HiPo versus LoPo, respectively (Additional file 1: Figure S4 A, B).

### HiPo scaffolds improve osteogenic differentiation and mineralization

**Fig. 4:**
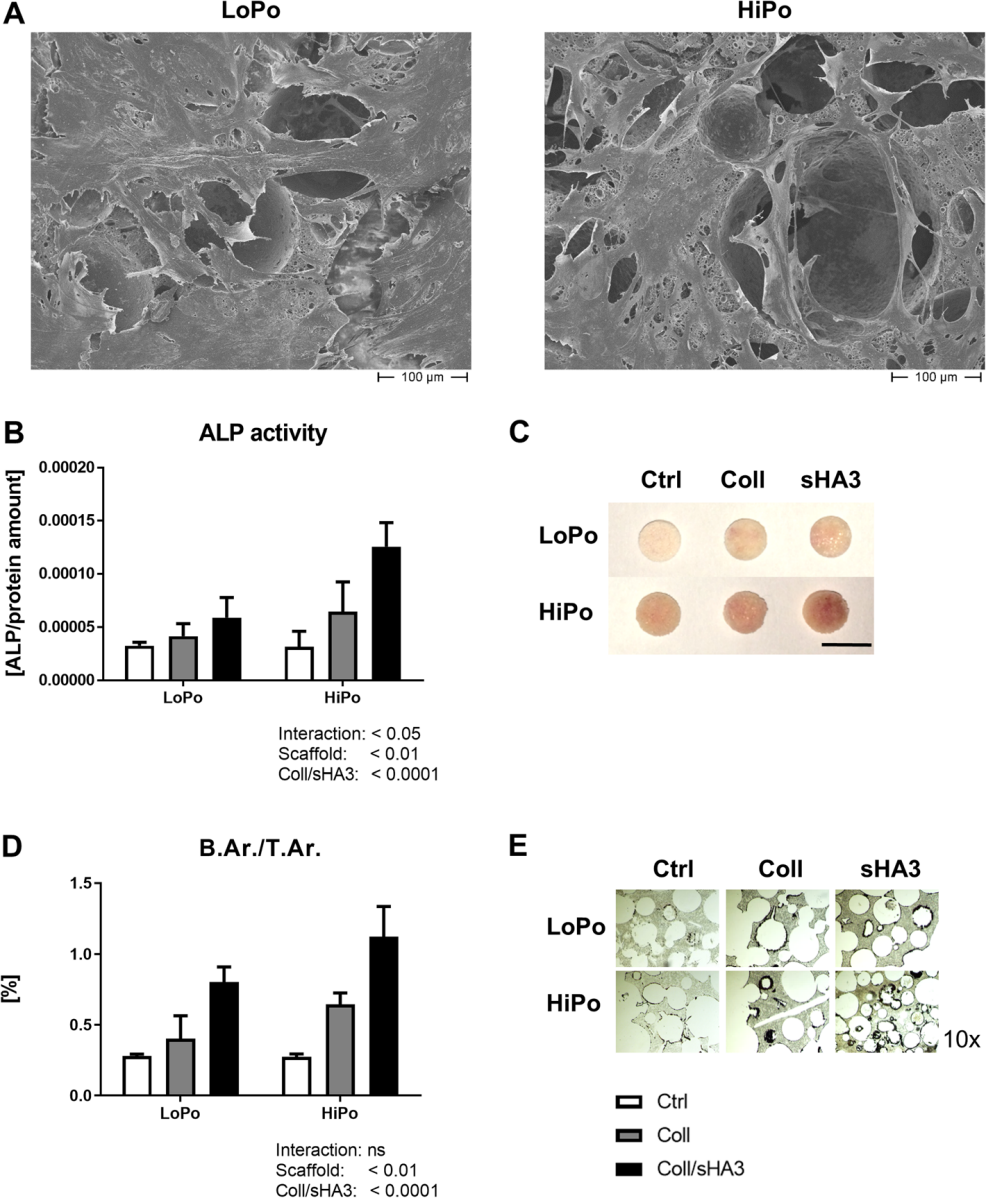
Characterization of cell culture on aECM-coated scaffolds. Human mesenchymal stem cells were cultured on LoPo and HiPo (low and high porosity, respectively) scaffolds for a time period of up to 18 days. **a** Representative scanning electron microscopy pictures of hMSC on LoPo (left) and HiPo (right) scaffolds were taken after 3 days of cultivation. **b** Cell functionality was quantified via the expression of the early osteogenic marker alkaline phosphatase (ALP) after seven days in culture by ALP quantification and **c** ALP staining. **d** + **e** The amount of synthesized mineralized matrix was measured by *von Kossa* staining on the histologic level. **c** ALP staining (red = ALP expression, scale bar = 5 mm) and **e** staining of mineralized bone material in scaffold pores (black = mineralized matrix). Data represent the mean ± SD. Statistical analysis was performed by two-way ANOVA for the effect of LoPo and HiPo scaffolds

For a comprehensive analysis, effects on cells seeded on LoPo and HiPo scaffolds were investigated at early (d3), intermediate (d7) and late (d18) stages of cell differentiation on the scaffolds. Initially, no marked differences could be observed between the two scaffold formulations. SEM pictures prepared after 3 days show hMSC with a wide spread and elongated morphology and cells spanning over pores, forming large confluent cell layers on either material (Fig. 4a). However, distinct differences were detected for their cell adhesion potential. When analyzed 24 h after cell seeding scaffolds prepared with a lower pore size retained more cells than HiPo scaffolds (Additional file 1: Figure S5A + D). However, cell numbers increased significantly faster on HiPo (Additional file 1: Figure S5C + F), reaching an equal cell density at day 3 (Additional file 1: Figure S4B + E).

The expression of ALP is an early marker of osteoblastic differentiation, which was shown to be enhanced by sGAG [14]. Here, sHA3 elevated ALP activity at day 7 on LoPo scaffolds about 82% and almost 3-fold on HiPo scaffolds which results in a total increase of ALP activity about 112% from LoPo to HiPo scaffolds (Fig. 4b, c).

The cell viability on LoPo and HiPo scaffolds did not differ after 14 days in culture (data not shown). Only on purely collagen-coated scaffolds the cell viability was increased in comparison with uncoated and coll/sHA3 controls. After 18 days cultivation, the synthesis of mineralized matrix in scaffold pores was analyzed by histology (Fig. 4d, e). Coating with collagen only (coll) increased the B.Ar./T.Ar. about 51% and coll/sHA3 coating about 107% on LoPo scaffolds. On HiPo scaffolds, coll coating enhanced mineralization about 71% and coll/sHA3 about 265%. The increase of matrix mineralization from LoPo to HiPo scaffolds after coll/sHA3 coating was 53%.

Similar results were obtained with pre-osteoblastic MC3T3-E1 cells that were cultured on HiPo scaffolds. After initially different seeding efficiencies no differences could be observed for the cell viability due to the scaffold type or coating (Additional file 1: Figure S6 A, B). In addition, the ALP activity and matrix mineralization were increased by coll/sHA3 coating and further enhanced by HiPo scaffolds (Additional file 1: Figure S6 C-F).

## Discussion

In this study, we improved the osteogenic potential of TriLA scaffolds to further increase their suitability for the application in individuals with impaired bone regeneration like in osteoporosis and diabetes patients. We achieved this by optimizing the pore size and distribution and a coating with sHA3-rich aECM. Our in vitro data on murine and human osteoblasts show that the elevation of pore size increased the osteogenic potential of osteoblasts.

The TriLA-platform of macromers was designed as a biodegradable, cross-polymerizing material to facilitate bone formation in critically sized defects. The initially developed formulation resulted in a mechanically resilient material, featuring a compressive modulus of 27.2 MPa that is comparable to values at the low end of the range of compressive modulus reported for trabecular bone [33]. When applied in a diabetic rat model [19], we observed bone healing, however at an insufficient slow rate. In the course of this experiment, we observed that the initial formulation is characterized by a low average pore size of less than 150 μm and limited interconnectivity. This restricts the mobility of the resident bone cells mainly to the scaffold surface and may have been decisive for their incomplete bone healing properties. To this end, we fine-tuned the parameters of the macromer cross-linking process to achieve a more favorable pore size for bone healing.

The use of lipids in solid lipid templating (SLT) for the generation of macroporous scaffolds has distinct advantages over techniques such as the use of salt- or sugar-based porogens. The melting of the lipid at cross-linking temperatures and their immiscibility with the polymer phase allows for the generation of a continuous, interconnected pore structure. Furthermore, the solubility of the lipids in a lipophilic solvent enables the exclusion of water from the temperature-elevated leaching process, preventing premature hydrolysis of the ester bonds between the building blocks of the TriLA macromer.

It was found to be impossible to increase the lipid to macromer ratio independently from the macromer concentration to further increase porosity. Increasing the lipid amount without tuning macromer concentration resulted in scaffold cylinders that displayed an uneven structure with major defects. The series of experiments indicated that porosity of the constructs (reference formulation) could not be significantly increased while maintaining stability and structural integrity with this method. Other available parameters to potentially improve pore network structure, however, were the lipid particle composition and the reaction temperature. By increasing the content of the lower melting lipid component, the resulting particles will melt at a lower temperature, as previous work with triglyceride lipids demonstrated [28, 34]. The melting rate of a substance is proportional to the difference in its melting point and the surrounding temperature and thus the energy available to overcome the melting enthalpy. As such, both the lowering of the lipid melting temperature and the raise in reaction temperature during cross-linking increases the volume of molten lipid at a given time point. This creates a pore network with higher pore sizes and bigger pore connections at the optimized conditions.

We achieved a mean pore size of about 210 μm, with a broad distribution of pore sizes ranging from about 50 μm up to 400 μm, and a relevant fraction exceeding 300 μm with the optimized TriLA scaffold formulation. As a consequence of the increased pore size, a decrease in compressive modulus to 20.3 MPa, which remains at low end of the range reported for the compressive modulus of trabecular bone, has to be accepted [33].

Due to the melting of the lipid and the formation of the continuous molten phase, the ultimate size of the pores in the scaffold is smaller than that of the lipid particles used for templating. This is in contrast to techniques that employ salt or sugar crystals as porogens, in which the final pore size is more consistent with the size of the porogens [35]. On the other hand, use of a porogen that remains in a solid state throughout the templating process limits the pore interconnectivity to the points where porogen particles touch while the continuous molten phase allows for the formation of interconnections between particles that are not direct adjacent to each other. This discrepancy in porogen size and final pore size is hence inherent to the processing technique and needs to be accounted for during scaffold production.

The achieved pore sizes of our TriLA scaffolds are comparable to other bone tissue engineering scaffolds published in literature, which were produced from similar poly(α-hydroxy acid)-based materials. Schardosim et al. could produce PLGA-based nanocomposite scaffolds with pore sizes ranging from 60 to 380 μm by freeze-casting [36] while Grémare et al. 3D-printed regular PLA-scaffolds with pore sizes from 150 to 250 μm using melt extrusion [37]. Working with a non-crosslinked poly (lactic acid)/PEG-based material, Bhaskar et al. generated porous scaffolds with a pore size between 0 and 300 μm by sugar leaching, with most of the pores being in the 100 to 200 μm range, and an overall porosity of about 60% [23]. Using PLGA-based scaffolds generated by our solid lipid templating approach, in vivo bone ECM formation was best with scaffolds made with lipid particles ranging from 300 to 500 μm in size [27].

High-sulfated GAGs such as sHA3 and sCS3 have been shown to increase osteogenic differentiation capacity of cell lines as well as murine and human MSC while concurrently reducing osteoclastogenesis in vitro [12, 14, 19, 38–40]. This results in an elevated matrix production by osteoblasts while bone resorption by osteoclasts is reduced, which is an ideal combination for bone regeneration. First in vivo studies already indicated an improved new bone formation induced by sGAGs. Hydrogels incorporating cross-linked chondroitin sulfate show mineralization with calcium phosphates both in vitro and in vivo [41]. Coatings of dental titanium implants with collagen and sGAGs increase the bone-implant contact and the peri-implant bone formation in maxillary bone of minipigs [42, 43]. In addition, collagen scaffolds enriched with LMW-HA improve the bone formation in calvarial critical-size defects in rodents [44]. In accordance, the gap size of a critical-size femur defect in rats decreases by coating titanium-coated polyetheretherketone plates with collagen and sGAGs by improving endochondral ossification [20]. The high sulfation of GAGs lead to an increased recruitment of osteoblastic pre-cursor cells [39], an improved cell adhesion [45], reduced inflammatory reactions by macrophages [46, 47], affects endothelial cell activation [48, 49], and binds cell growth factors such as BMP 2 and 4, and transforming growth factor β1 (TGF-β1) [17, 50] or sclerostin, an inhibitor of the osteogenic Wnt signaling pathway [18, 19]. Recently, we analyzed the bone formation of a sub-critical femur defect in diabetic rats characterized by a delayed fracture healing [19, 51]. TriLA (LoPo) scaffolds coated with coll/sHA3 were inserted into the defect area resulting in enhanced bone formation in diabetic rats that elevated their healing level up to that of healthy wild type controls. This was mediated by increased osteoblast differentiation and a prolonged immobilization of sclerostin by sHA3 [19]. Because the defect filling remained incomplete even under improved conditions provided by the sGAG coatings, we investigated the HiPo TriLA scaffolds. Here, we were able to coat HiPo scaffolds with an increased amount of collagen and sHA3 resulting in an increased osteogenesis as well as mineralization capacity of a murine cell line and human MSC in vitro. This is in line with previous findings demonstrating a positive effect of the coll/sHA3 coating on bone formation in vivo [19].

Work on a non-crosslinked poly (lactic acid)/PEG-based material showed that an increase in average pore size while keeping porosity constant results in an improved growth and proliferation of osteoblastic bone cells [23]. A similar improvement of cellular attachment and proliferation with increasing pore size and collagen incorporation was found in a previous work with poly(α-hydroxy acid)-based and collagen/GAG-based porous scaffolds [52–54]. This indicates that materials chemically similar to the individual components of the coated, cross-polymerized scaffolds examined in this work, displayed a comparable effect of improved cellular response with increased pore size.

## Conclusion

We were able to optimize the pore size of TriLA scaffolds and thereby maximized the coating amount of osteo-inductive aECM. This resulted in an increased osteogenesis of osteoblast precursor cells and elevated formation of new bone matrix in vitro. For future studies, a higher amount of osteoinductive aECM with sGAGs could be targeted to bone defect areas to enhance new bone formation under normal and compromised conditions like in osteoporosis and diabetes.

## Supplementary information

**Additional file 1: Figure S1.** Investigation of macromer concentration and lipid content. **Figure S2.** Investigation of lipid composition. **Figure S3.** Investigation of reaction temperature and particle size, in both this box detailing the contents of Additional file 1 and in Additional file 1 itself. **Figure S4.** Investigation of long-term stability of aECM coating on scaffolds. **Figure S5.** HiPo scaffolds improve cell proliferation of human mesenchymal stromal cells. **Figure S6.** HiPo scaffolds further improve osteogenic differentiation and mineralization of pre-osteoblastic MC3T3-E1 cells.

## Data Availability

The datasets used and/or analyzed during the current study are available from the corresponding author on reasonable request.

## Abbreviations

aECM: Artificial ECM
ALP: Alkaline Phosphatase
ANOVA: Analysis of variance
BCA: Bicinchoninic acid
BMP-2: Bone morphogenic protein-2
CaP: Calcium phosphate
Coll: Collagen type I
CS: Chondroitin sulfate
ddH_2_O: Double distilled water
DS: Degree of sulfation, average number of sulfate groups per repeating disaccharide unit
ECM: Extracellular matrix
FI: Fluorescence intensity
Fig: Figure
GAGs: Glycosaminoglycans
GPC: Gel permeation chromatography
HA: Hyaluronan
HiPo: Scaffolds with higher pore size
hMSCs: Human mesenchymal stem cells
LA: Lactic acid
LMW-HA: Low molecular weight HA
LoPo: Scaffolds with lower pore size
Mn: Number-average molecular weight
Mw: Molecular weight
OPA: *o*-phthaldialdehyde
OPG: Osteoprotegerin
PBS: Phosphate-buffered saline
PD: Polydispersity index
PEG: Polyethylene glycol
PEG-MA: PEG-monomethacrylate
PFA: Paraformaldehyde
PLA: Poly (lactic acid)
PLGA: Poly (lactic acid-*co*-glycolic acid)
RI: Refraction index
SD: Standard deviation
SEM: Scanning electron microscopy
sHA3: High-sulfated hyaluronan, degree of sulfation: 3
SLT: Solid lipid templating
TES: 2-[[1,3-dihydroxy-2-(hydroxymethyl)propan-2-yl]amino] ethanesulfonic acid
TGF-β1: Transforming growth factor β1
TriLA: Three-armed lactic acid-based scaffolds
