# Supplementary material for: Increased pore size of scaffolds improves coating efficiency with sulfated hyaluronan and mineralization capacity of osteoblasts
Source: Biomater Res. 2019 Dec 18;23:26. doi: 10.1186/s40824-019-0172-z (PMC6921484; doi:10.1186/s40824-019-0172-z)
Supplement: Supplementary file 1 — Additional file 1: Figure S1. Investigation of macromer concentration and lipid content. Figure S2. Investigation of lipid composition. Figure S3. Investigation of reaction temperature and particle size, in both this box detailing the contents of Additional file 1 and in Additional file 1 itself. Figure S4. Investigation of long-term stability of aECM coating on scaffolds. Figure S5. HiPo scaffolds improve cell proliferation of human mesenchymal stromal cells. Figure S6. HiPo scaffolds further improve osteogenic differentiation and mineralization of pre-osteoblastic MC3T3-E1 cells. [file 40824_2019_172_MOESM1_ESM.pptx]

## Slide 1
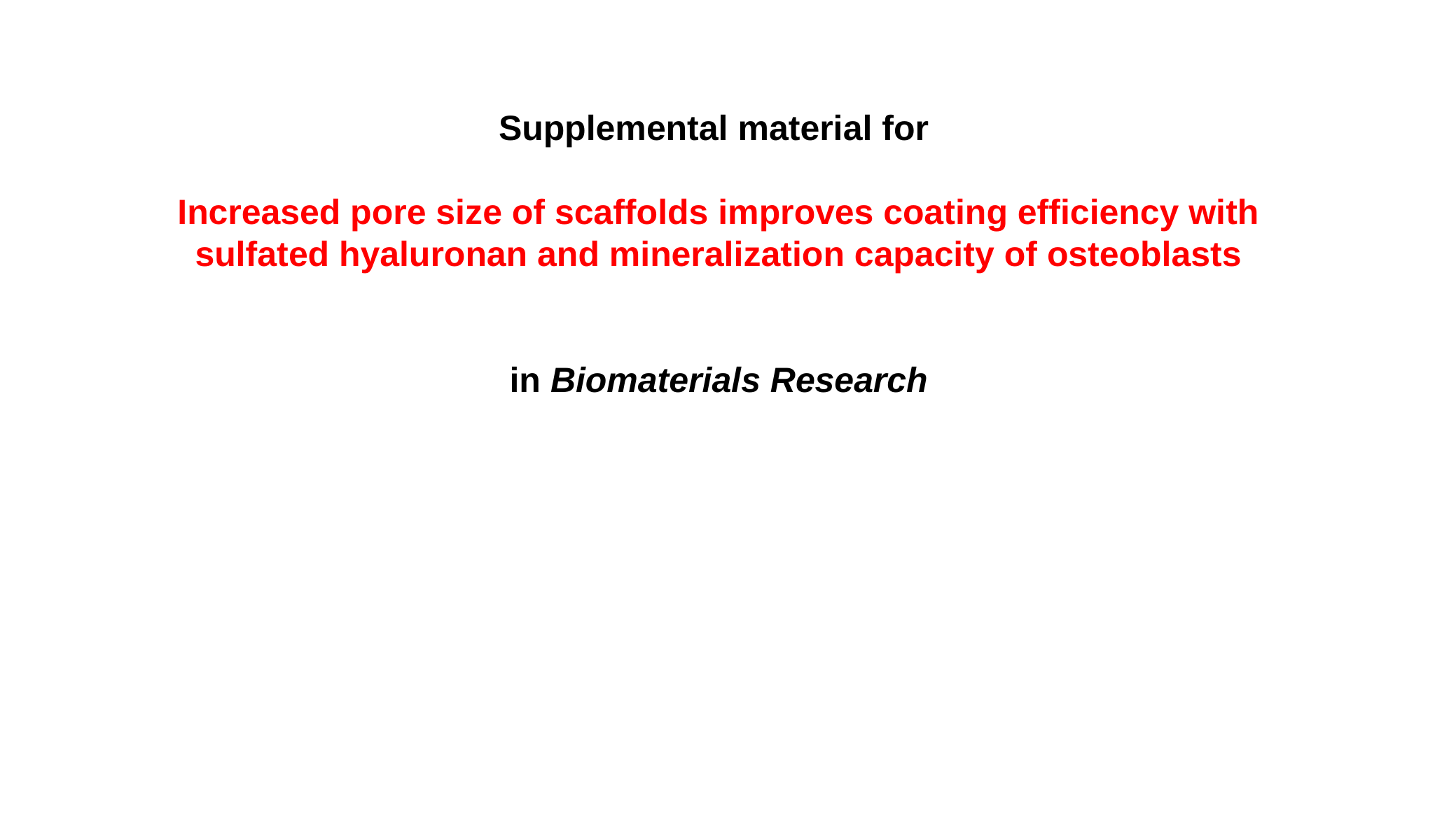

Supplemental material for
Increased pore size of scaffolds improves coating efficiency with sulfated hyaluronan and mineralization capacity of osteoblasts
in Biomaterials Research

## Slide 2
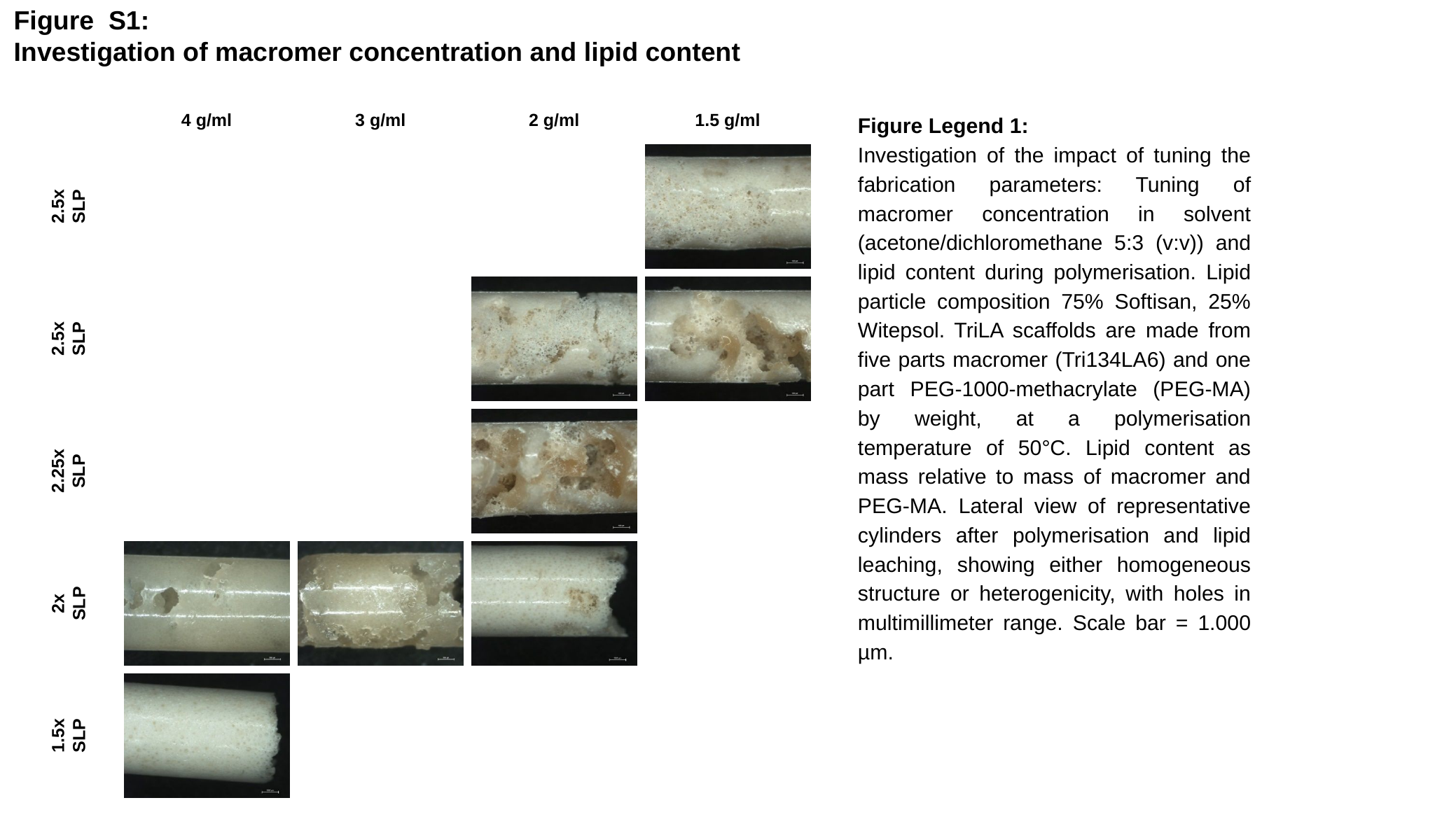

Figure S1:
Investigation of macromer concentration and lipid content
4 g/ml
3 g/ml
2 g/ml
1.5 g/ml
2.5x
SLP
2.5x
SLP
2.25x
SLP
2x
SLP
1.5x
SLP
Figure Legend 1:
Investigation of the impact of tuning the fabrication parameters: Tuning of macromer concentration in solvent (acetone/dichloromethane 5:3 (v:v)) and lipid content during polymerisation. Lipid particle composition 75% Softisan, 25% Witepsol. TriLA scaffolds are made from five parts macromer (Tri134LA6) and one part PEG-1000-methacrylate (PEG-MA) by weight, at a polymerisation temperature of 50°C. Lipid content as mass relative to mass of macromer and PEG-MA. Lateral view of representative cylinders after polymerisation and lipid leaching, showing either homogeneous structure or heterogenicity, with holes in multimillimeter range. Scale bar = 1.000 µm.

## Slide 3
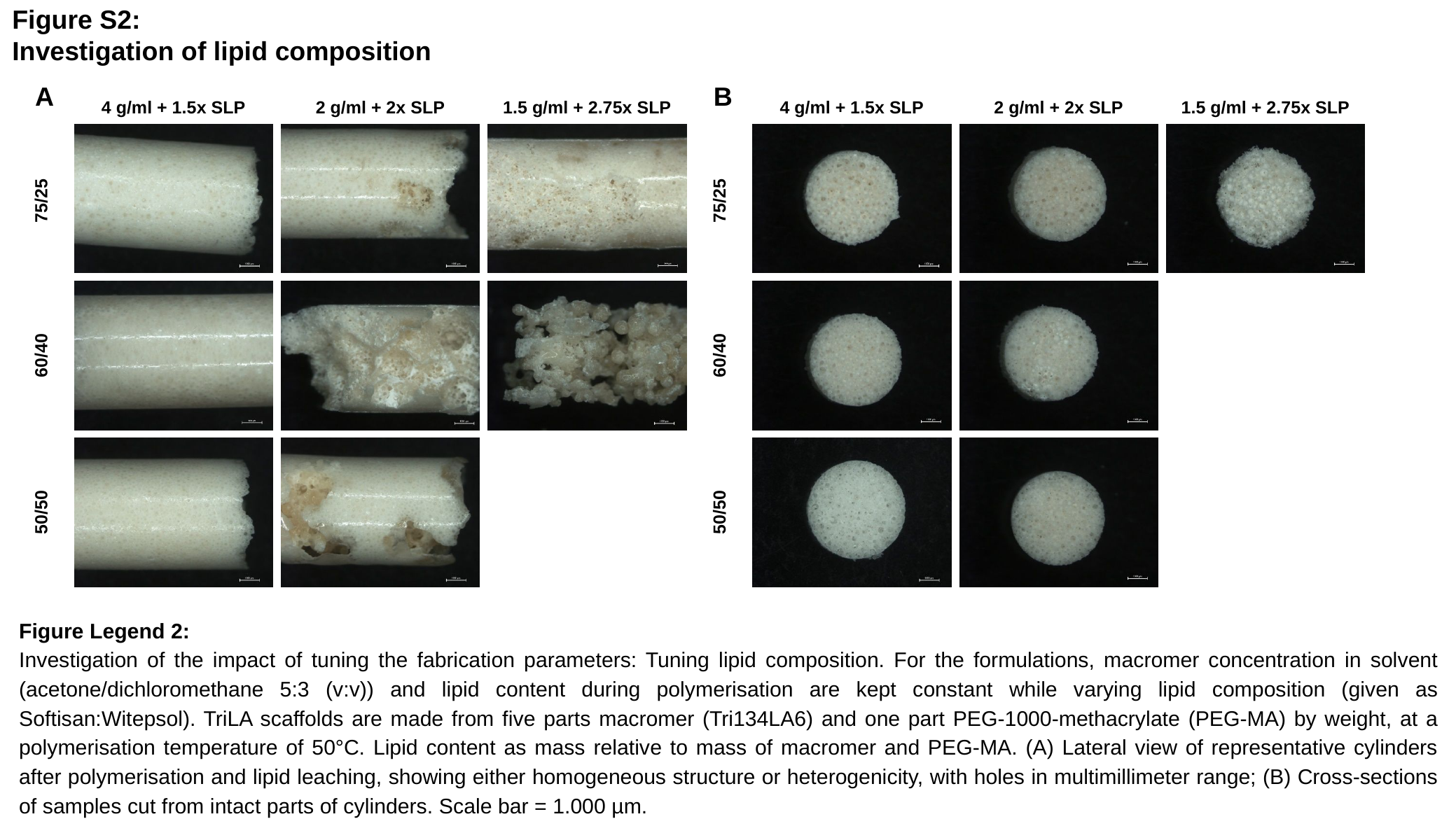

Figure S2:
Investigation of lipid composition
A
4 g/ml + 1.5x SLP
2 g/ml + 2x SLP
1.5 g/ml + 2.75x SLP
75/25
60/40
50/50
B
4 g/ml + 1.5x SLP
2 g/ml + 2x SLP
1.5 g/ml + 2.75x SLP
75/25
60/40
50/50
Figure Legend 2:
Investigation of the impact of tuning the fabrication parameters: Tuning lipid composition. For the formulations, macromer concentration in solvent (acetone/dichloromethane 5:3 (v:v)) and lipid content during polymerisation are kept constant while varying lipid composition (given as Softisan:Witepsol). TriLA scaffolds are made from five parts macromer (Tri134LA6) and one part PEG-1000-methacrylate (PEG-MA) by weight, at a polymerisation temperature of 50°C. Lipid content as mass relative to mass of macromer and PEG-MA. (A) Lateral view of representative cylinders after polymerisation and lipid leaching, showing either homogeneous structure or heterogenicity, with holes in multimillimeter range; (B) Cross-sections of samples cut from intact parts of cylinders. Scale bar = 1.000 µm.

## Slide 4
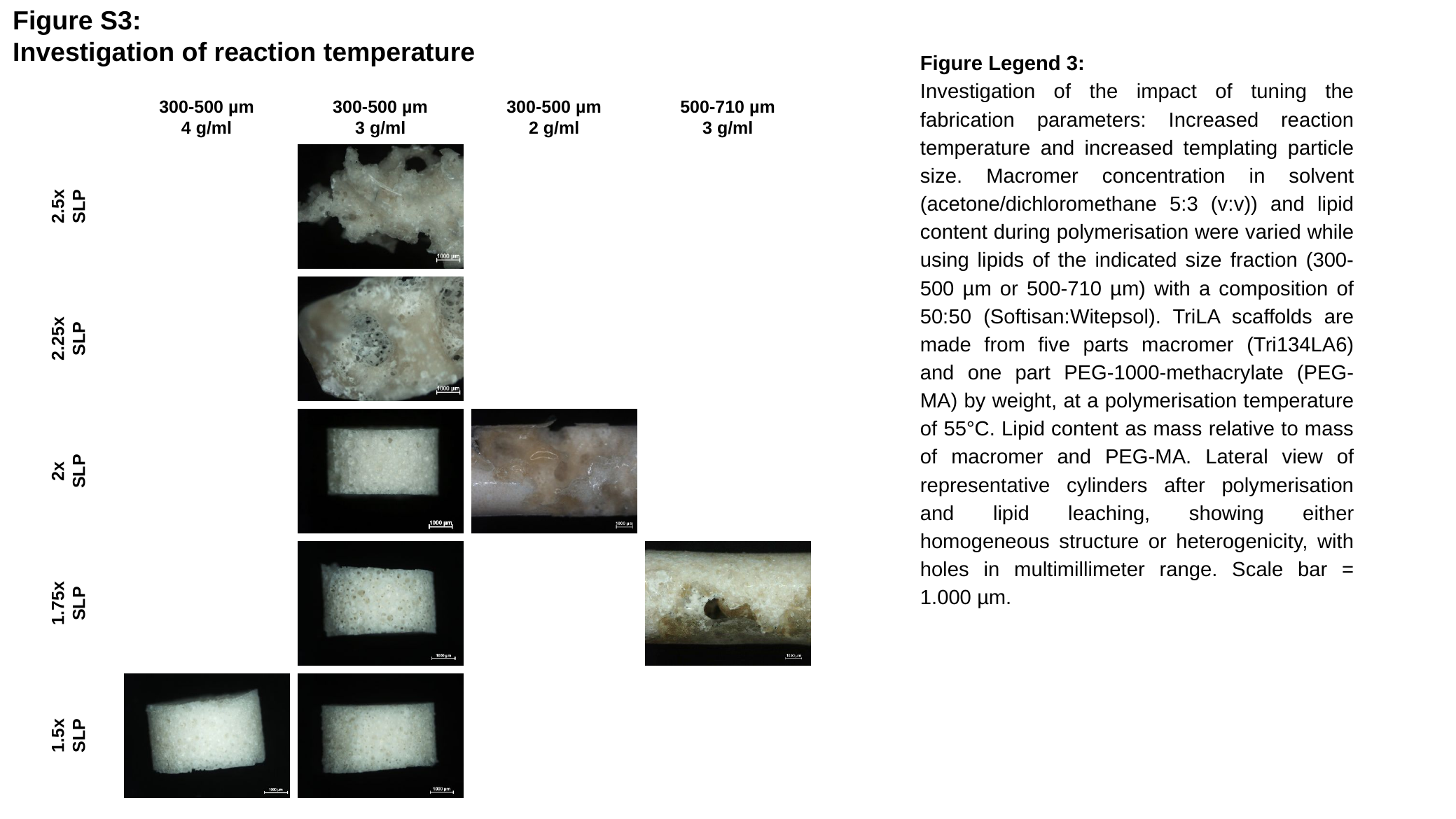

Figure S3:
Investigation of reaction temperature
Figure Legend 3:
Investigation of the impact of tuning the fabrication parameters: Increased reaction temperature and increased templating particle size. Macromer concentration in solvent (acetone/dichloromethane 5:3 (v:v)) and lipid content during polymerisation were varied while using lipids of the indicated size fraction (300-500 µm or 500-710 µm) with a composition of 50:50 (Softisan:Witepsol). TriLA scaffolds are made from five parts macromer (Tri134LA6) and one part PEG-1000-methacrylate (PEG-MA) by weight, at a polymerisation temperature of 55°C. Lipid content as mass relative to mass of macromer and PEG-MA. Lateral view of representative cylinders after polymerisation and lipid leaching, showing either homogeneous structure or heterogenicity, with holes in multimillimeter range. Scale bar = 1.000 µm.
300-500 µm
4 g/ml
300-500 µm
3 g/ml
300-500 µm
2 g/ml
500-710 µm
3 g/ml
2.5x
SLP
2.25x
SLP
2x
SLP
1.75x
SLP
1.5x
SLP

## Slide 5
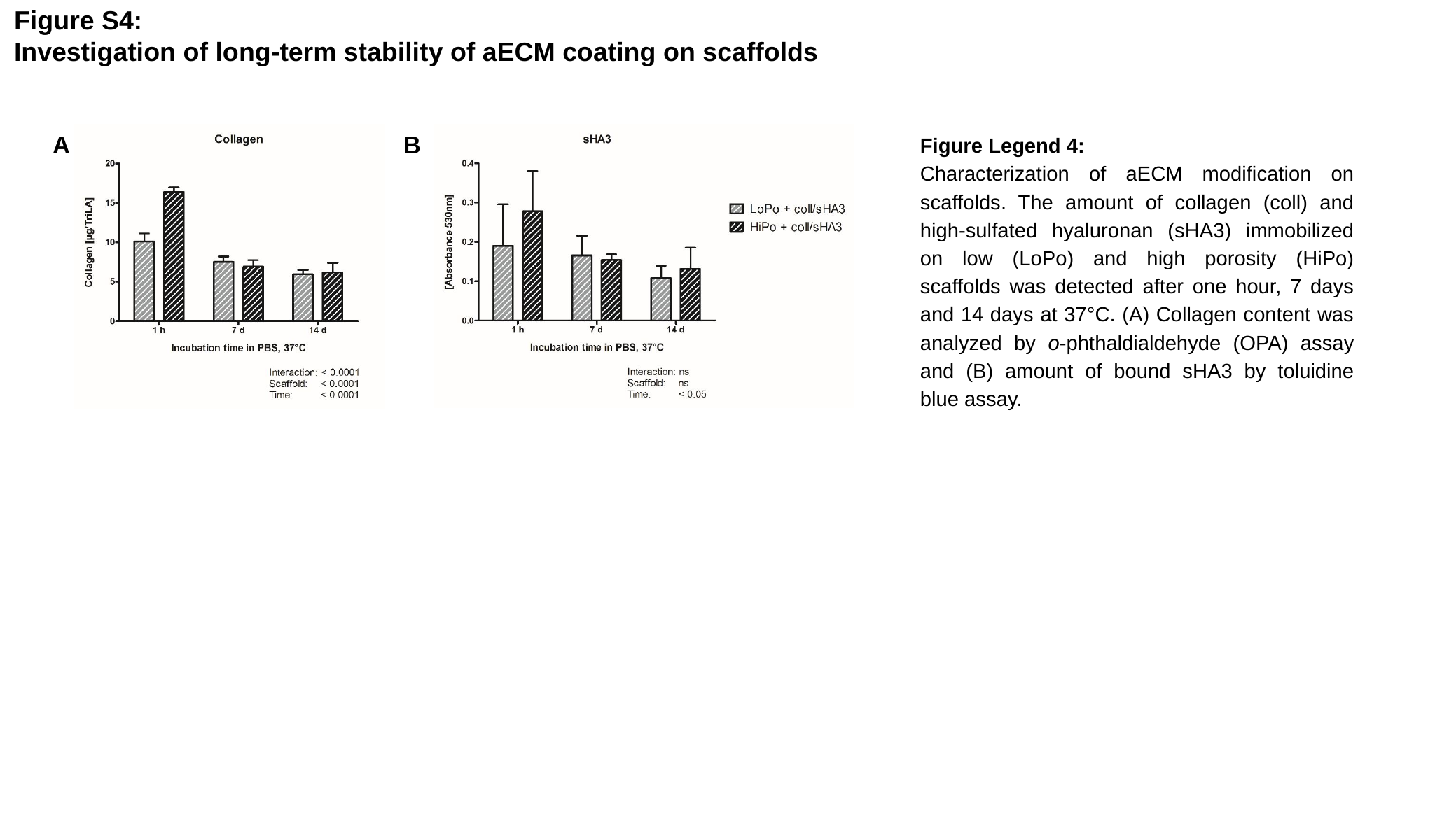

Figure S4:
Investigation of long-term stability of aECM coating on scaffolds
A
B
Figure Legend 4:
Characterization of aECM modification on scaffolds. The amount of collagen (coll) and high-sulfated hyaluronan (sHA3) immobilized on low (LoPo) and high porosity (HiPo) scaffolds was detected after one hour, 7 days and 14 days at 37°C. (A) Collagen content was analyzed by o-phthaldialdehyde (OPA) assay and (B) amount of bound sHA3 by toluidine blue assay.

## Slide 6
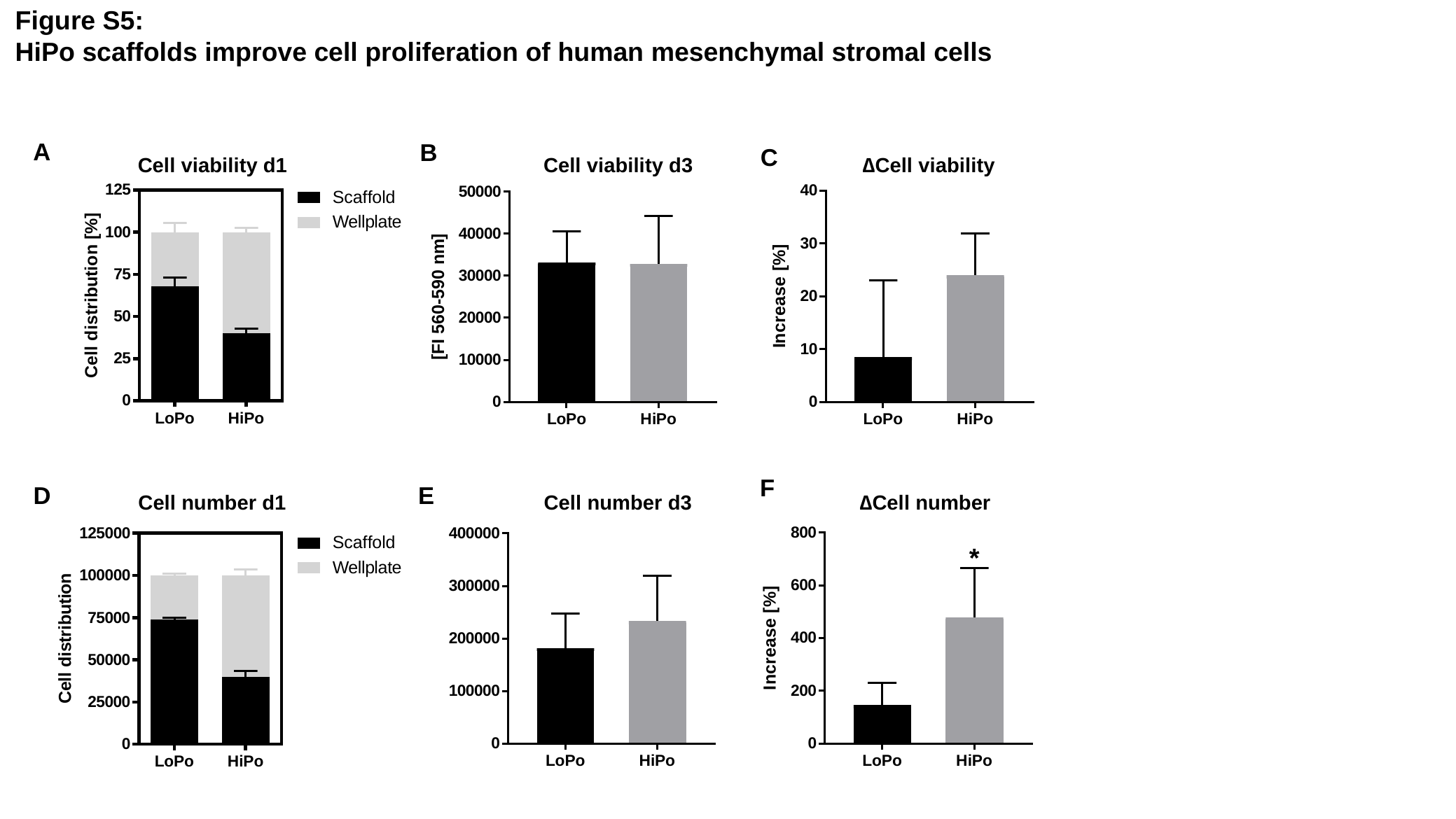

Figure S5:
HiPo scaffolds improve cell proliferation of human mesenchymal stromal cells
A
B
C
∆Cell viability
Cell viability d3
Cell viability d1
F
D
E
∆Cell number
Cell number d3
Cell number d1

## Slide 7
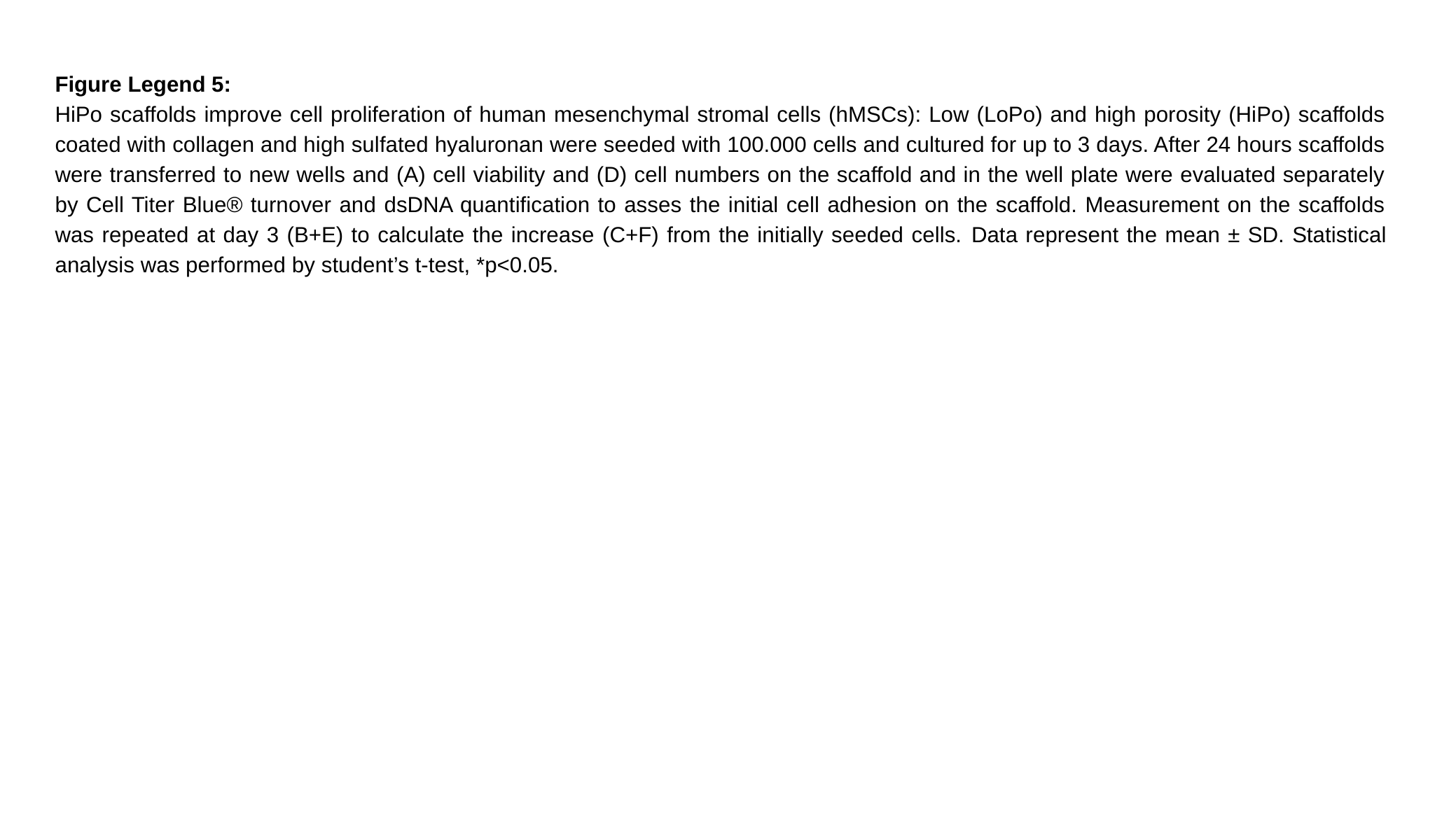

Figure Legend 5:
HiPo scaffolds improve cell proliferation of human mesenchymal stromal cells (hMSCs): Low (LoPo) and high porosity (HiPo) scaffolds coated with collagen and high sulfated hyaluronan were seeded with 100.000 cells and cultured for up to 3 days. After 24 hours scaffolds were transferred to new wells and (A) cell viability and (D) cell numbers on the scaffold and in the well plate were evaluated separately by Cell Titer Blue® turnover and dsDNA quantification to asses the initial cell adhesion on the scaffold. Measurement on the scaffolds was repeated at day 3 (B+E) to calculate the increase (C+F) from the initially seeded cells. Data represent the mean ± SD. Statistical analysis was performed by student’s t-test, *p<0.05.

## Slide 8
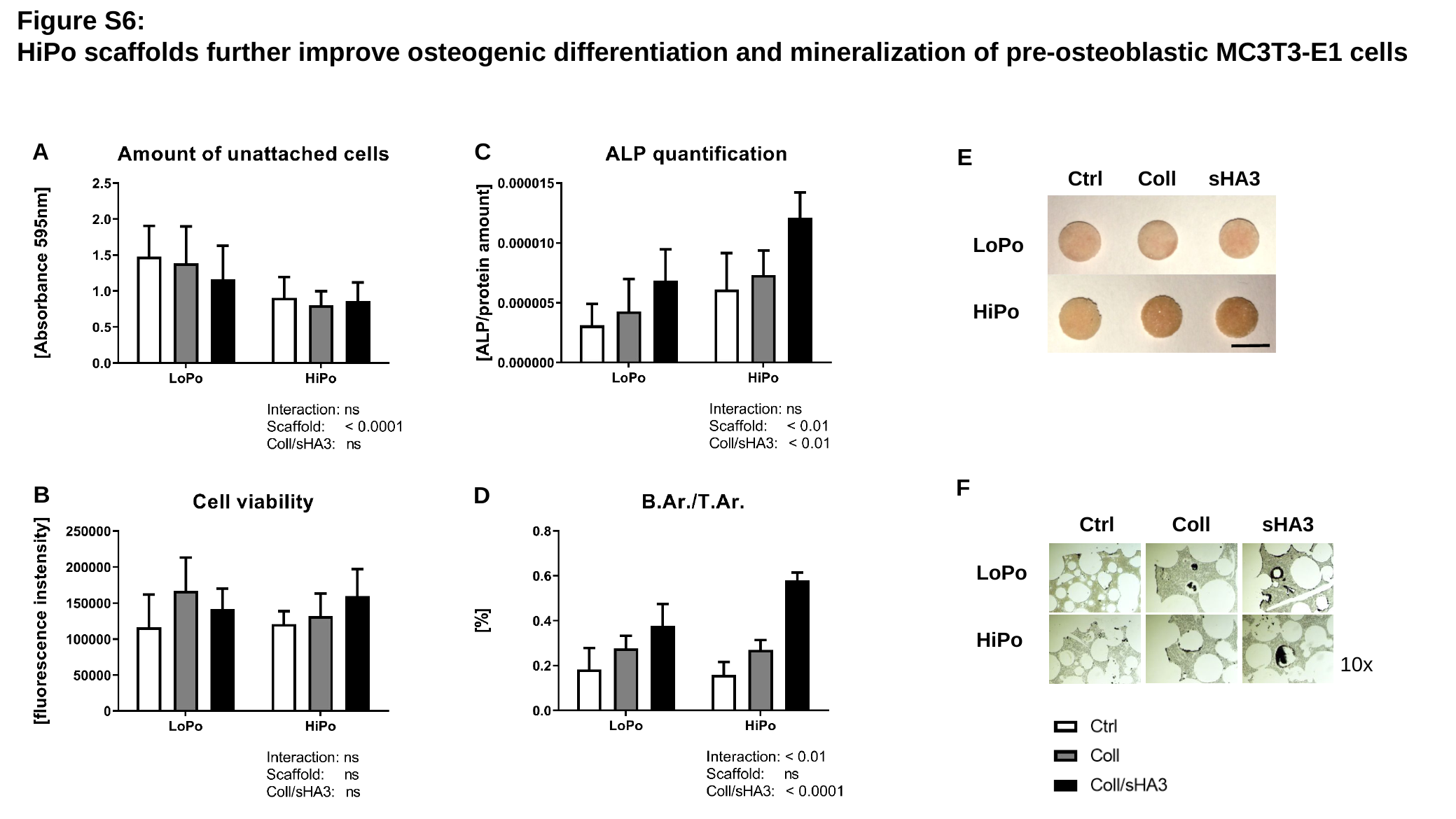

Figure S6:
HiPo scaffolds further improve osteogenic differentiation and mineralization of pre-osteoblastic MC3T3-E1 cells
A
C
E
Ctrl
Coll
sHA3
LoPo
HiPo
F
B
D
Ctrl
Coll
sHA3
LoPo
HiPo
10x

## Slide 9
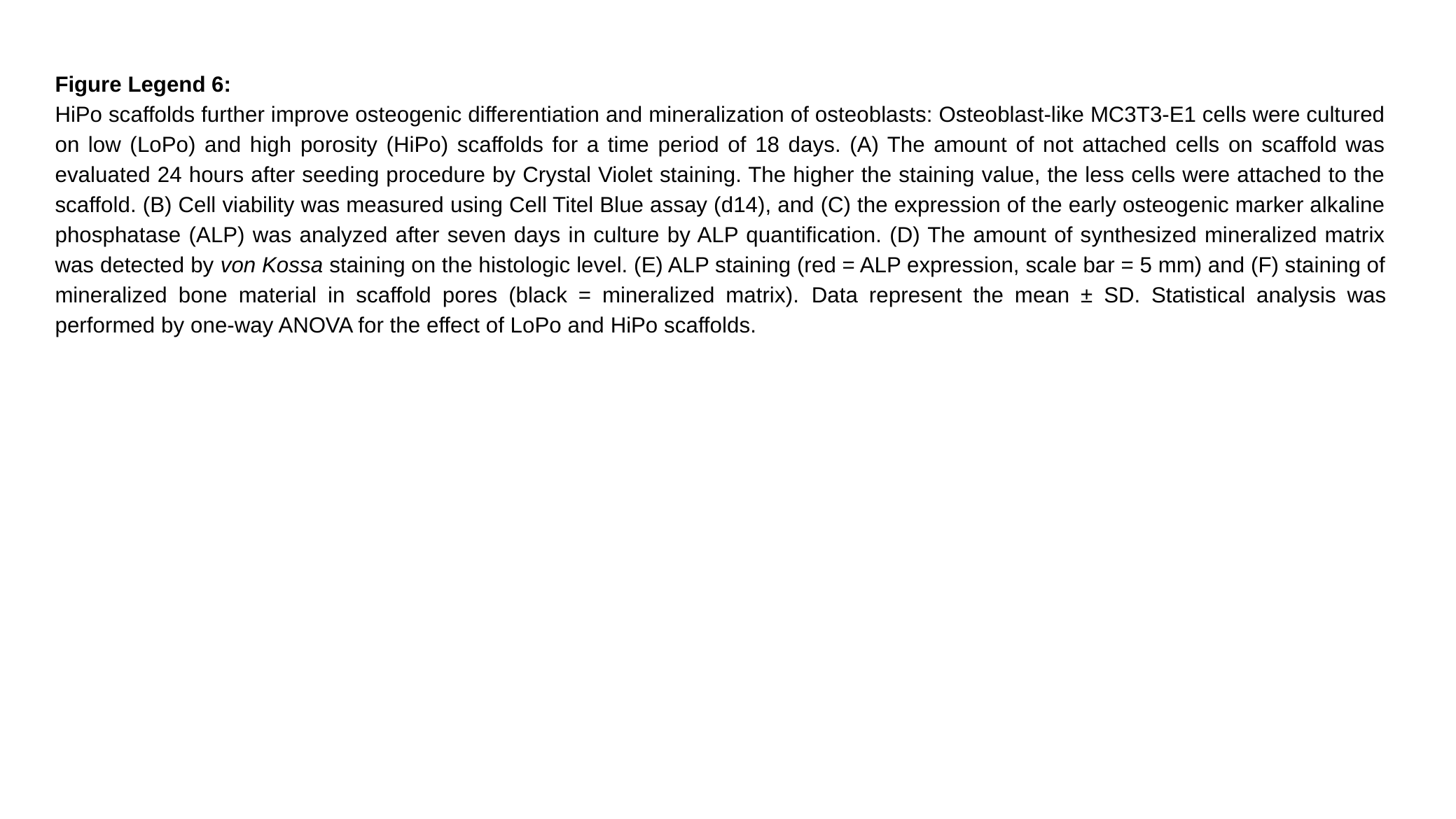

Figure Legend 6:
HiPo scaffolds further improve osteogenic differentiation and mineralization of osteoblasts: Osteoblast-like MC3T3-E1 cells were cultured on low (LoPo) and high porosity (HiPo) scaffolds for a time period of 18 days. (A) The amount of not attached cells on scaffold was evaluated 24 hours after seeding procedure by Crystal Violet staining. The higher the staining value, the less cells were attached to the scaffold. (B) Cell viability was measured using Cell Titel Blue assay (d14), and (C) the expression of the early osteogenic marker alkaline phosphatase (ALP) was analyzed after seven days in culture by ALP quantification. (D) The amount of synthesized mineralized matrix was detected by von Kossa staining on the histologic level. (E) ALP staining (red = ALP expression, scale bar = 5 mm) and (F) staining of mineralized bone material in scaffold pores (black = mineralized matrix). Data represent the mean ± SD. Statistical analysis was performed by one-way ANOVA for the effect of LoPo and HiPo scaffolds.
